# Evaluating Large Language Model‐Assisted Emergency Triage: A Comparison of Acuity Assessments by GPT‐4 and Medical Experts

**DOI:** 10.1111/jocn.17490

**Published:** 2024-11-28

**Authors:** Gal Ben Haim, Mor Saban, Yiftach Barash, David Cirulnik, Amit Shaham, Ben Zion Eisenman, Livnat Burshtein, Orly Mymon, Eyal Klang

**Affiliations:** ^1^ Department of Emergency Medicine Sheba Medical Center Ramat‐Gan Israel; ^2^ Faculty of Medical and Health Sciences, Tel Aviv University Tel Aviv Israel; ^3^ Division of Diagnostic Imaging Sheba Medical Center Tel Hashomer Israel; ^4^ DeepVision Lab Sheba Medical Center Tel Hashomer Israel; ^5^ The Division of Data‐Driven and Digital Medicine (D3M) Icahn School of Medicine at Mount Sinai New York USA

**Keywords:** AI in healthcare, clinical decision support, Emergency Severity Index, GPT‐4, large language model, patient triage

## Abstract

**Aim:**

To evaluate the accuracy of the Emergency Severity Index (ESI) assignments by GPT‐4, a large language model (LLM), compared to senior emergency department (ED) nurses and physicians.

**Method:**

An observational study of 100 consecutive adult ED patients was conducted. ESI scores assigned by GPT‐4, triage nurses, and by a senior clinician. Both model and human experts were provided the same patient data.

**Results:**

GPT‐4 assigned a lower median ESI score (2.0) compared to human evaluators (median 3.0; *p* < 0.001), suggesting a potential overestimation of patient severity by the LLM. The results showed differences in the triage assessment approaches between GPT‐4 and the human evaluators, including variations in how patient age and vital signs were considered in the ESI assignments.

**Conclusion:**

While GPT‐4 offers a novel methodology for patient triage, its propensity to overestimate patient severity highlights the necessity for further development and calibration of LLM tools in clinical environments. The findings underscore the potential and limitations of LLM in clinical decision‐making, advocating for cautious integration of LLMs in healthcare settings.

**Reporting Method:**

This study adhered to relevant EQUATOR guidelines for reporting observational studies.

**Summary**

- GPT‐4 showed a tendency for over‐triage vs. human clinicians in emergency severity indexing.
- Differences in GenAI vs. experienced triage approaches highlight need for model calibration before scalable clinical use.
- Findings underscore the importance of ongoing evaluation and refinement of GenAI tools to ensure efficacy, safety, and resource optimization when integrated into healthcare systems.
- Subtle discrepancies in how GPT‐4 and experts consider age, vitals point to potential biases in current GenAI training approaches needing attention.
- Strategic alignment of GenAI capabilities with real‐world clinical complexities is vital as artificial intelligence assumes increasing roles across medical specialties.

## 1 Introduction

Triage is a core function in emergency departments worldwide whereby patients are prioritised according to acuity levels using validated triage scales (Zachariasse et al. 2019). The Emergency Severity Index (ESI) is one such widely adopted five‐level algorithm used internationally to categorise patient needs and direct care (Meral et al. 2024). The ESI categorises patients into five levels, with 1 representing the highest urgency (e.g., immediate life‐threatening condition) and 5 the lowest (e.g., non‐urgent, minor injury/illness). This triage score directly informs the speed of clinical evaluation, diagnostic workup and treatment interventions (Meral et al. 2024). Traditionally, ESI assignment has relied on the clinical judgement and experience of senior emergency nurses at the frontlines. However, variations in triage decisions between providers have also been reported (Sax et al. 2023; Zaboli et al. 2023).

This variability can be attributed to several factors. First, the subjective nature of the ESI criteria leaves room for individual interpretation and bias. Nurse experience, training and decision‐making heuristics can all influence how they evaluate a patient's presenting signs and symptoms (Hinson et al. 2019). Several cognitive factors could potentially lead nurses to assign ESI scores that do not fully capture the patient's true clinical status. For example, an initial impression (‘anchor’) of a patient as stable may bias a nurse towards a lower acuity score, even if later signs or symptoms indicate higher risk. Additionally, jumping to a quick diagnosis (‘premature closure’) without fully considering alternative possibilities could result in an acuity score that does not fully account for the patient's condition (Kuriyama, Urushidani, and Nakayama 2017).

Furthermore, environmental and organisational factors can impact ESI scoring consistency. Factors like staffing levels, patient volume, distractions and institutional policies may introduce additional variability in the triage process (Kuriyama, Urushidani, and Nakayama 2017). Inadequate support systems or feedback mechanisms can also contribute to inconsistencies in nurse‐scored ESI. Concurrently, artificial intelligence (AI) technologies, such as large language models (LLMs), are rapidly transforming healthcare through applications of machine learning and natural language processing (Akinci D'Antonoli et al. 2024; Lucas, Upperman, and Robinson 2024). LLMs have gained attention for their potential to augment clinical decision‐making given capabilities like multilingual understanding and generation when trained on vast text corpora (Liao et al. 2024).

For example, a recent study examined the potential for an LLM to replicate the triage decision‐making of experienced emergency nurses. The researchers compared the triage code assignments made by an LLM (Chat‐GPT 3.5) against those documented by senior nurses for actual patient encounters. While there was some concordance between the LLM and nurses, the nurses demonstrated significantly better performance in predicting important clinical outcomes like 72‐h mortality. This suggests current LLMs may still have limitations in accurately assessing patient acuity compared to experienced human clinicians. More development is needed before such tools can reliably replace clinical judgement in triage. Providing full statistical results would be more appropriate once the topic is further discussed in the methods section (Zaboli et al. 2024).

While prior research has demonstrated machine learning models' effectiveness in clinical prediction tasks, direct comparisons of different LLMs modalities' performance replicating real‐world medical roles relative to human experts remain limited (Johnson et al. 2023; Liao et al. 2024; The Lancet Digital Health 2023). If validated further, such AI‐powered triage tools could potentially help standardise the intake process and complement the clinical judgement of frontline staff. Such evaluation studies are imperative as LLMS integration into clinical workflows accelerates.

This study aims to provide a rigorous comparison by prospectively assessing the accuracy of GPT‐4, a state‐of‐the‐art generative AI (GenAI) model developed by OpenAI, in assigning ESIs against senior emergency nurses managing actual patients (Liao et al. 2024). GPT‐4 was selected for its advanced natural language processing abilities but its validity in replicating frontline medical decision‐making processes like triage has yet to be thoroughly validated (Nori et al. 2023). To do so, it discusses de‐identified real cases verbally with GPT‐4 and compares its triage classifications to both nurses' documentation and the final triage decisions made by senior emergency physicians, analysing concordance rates between the LLM system, nursing assessments and physician determinations. By directly evaluating an LLM's ESI assignments against experienced clinicians, insights into AI's current potentials and limitations for clinical support roles can be gleaned (Ito et al. 2023; Oztermeli and Oztermeli 2023). Moreover, acknowledging LLMs represent just one AI modality, the findings may inform appropriate integration and oversight considerations for different technologies.

## 2 Methods

### 2.1 Study Design and Population

This observational study was conducted on November 23, 2023 at the Chaim Sheba Medical Center in Tel‐Hashomer, a large university‐affiliated tertiary referral hospital located in the Tel Aviv metropolitan area of Israel. Annually, the Chaim Sheba Emergency Department sees over 225,000 patient visits, providing a relevant high‐volume setting to evaluate the performance of an LLM‐based triage system against human expert decision‐making. We examined a cohort of consecutive patients to ensure a representation of the typical emergency department demographic and clinical spectrum. This approach aimed to provide a realistic and varied set of patient scenarios for ESI assignment comparison.

The study population comprised of 100 consecutive adult patients, presenting to the emergency department within a 24‐h period. These patients reflected a wide range of ages, genders and medical conditions, mirroring the heterogeneity commonly encountered in emergency settings.

This study adhered to relevant Enhancing the QUAlity and Transparency of Health Research (EQUATOR) guidelines for reporting observational studies.

### 2.2 Data Collection

For each patient, we collected data from the electronic health record, including demographic information, chief complaints, vital signs and details from nurse anamnesis and their ESI given by the triage nurse. This data collection was carried out to capture the nuances of each patient's condition, which are important for accurate ESI assignment.

Three senior nurses, each with extensive experience in emergency care, independently assigned an ESI level to each patient case based on the collected data. Neither the physicians and nurses who assessed the cases nor the AI model knew what the original triage score was.

We then tasked GPT‐4 with assigning ESI levels to the same set of patients.

A fourth assignment of ESI was performed by an ED senior physician.

**FIGURE 1:**
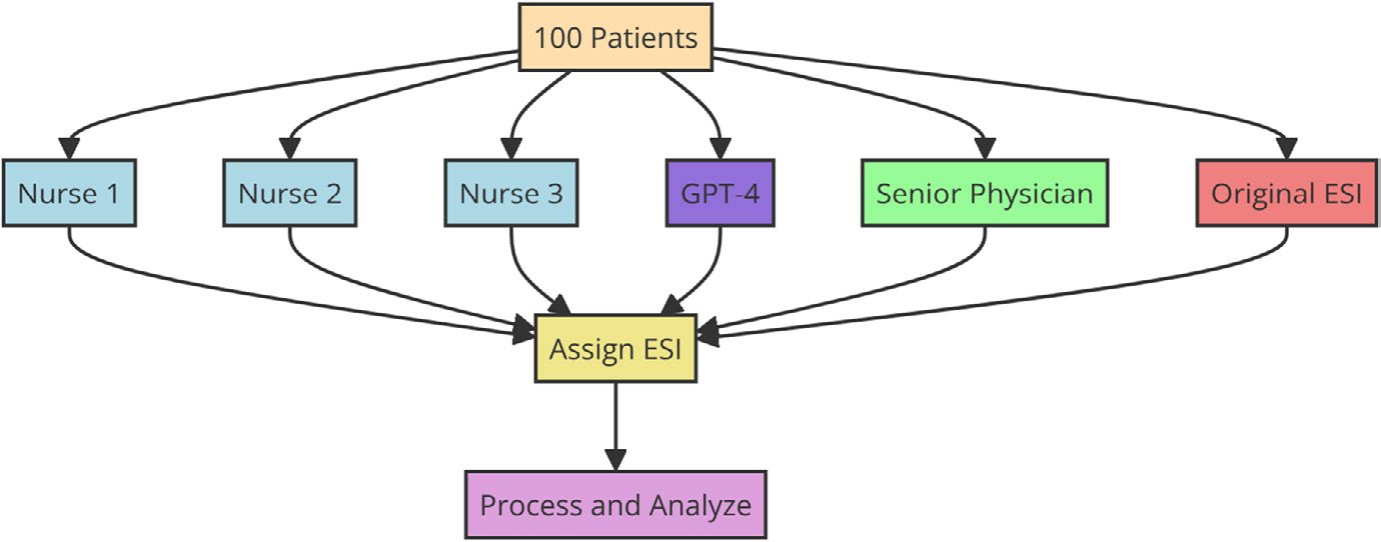
Study design – we compared ESI assignment between original ED ESI score, 3 senior nurses, an experienced ED physician and GPT‐4. [Colour figure can be viewed at wileyonlinelibrary.com]

Thus, each patient had four methods of ESI assigned scores:

(1) original ESI score assigned by the triage nurse, (2) three ESI scores assigned independently retrospectively by three seasoned emergency department nurses, (3) GPT‐4 assigned score and (4) experienced emergency department attending assigned ESI score (Figure 1).

Both the model and human experts were provided with the same patient data for comparison.

For all experiments, we used OpenAI's web interface to query GPT‐4. The prompt used for GPT‐4 was described as follows: ‘Can you please read this imaginary clinical note of an ER entry of an imaginary patient and give the proper ESI score to the patient? Please return only the numerical score, no other text’. Each query was done in a new instance to prevent data leakage.

### 2.3 Emergency Severity Index (ESI) Algorithm

The ESI is a five‐level triage algorithm used to assess patient severity in emergency departments (Agency for Healthcare Research and Quality n.d.; Platts‐Mills et al. 2010). The ESI helps prioritise patient care by identifying the severity of their condition and the resources needed for treatment. Level 1 represents the most urgent need for medical intervention, while Level 5 indicates the least urgency. The protocol is designed to facilitate quick and accurate decisions, guiding the triage nurse or practitioner in categorising patients efficiently:

Level 1: Immediate life‐saving intervention required.

Level 2: High risk of deterioration or signs of a severe condition.

Level 3: Requires multiple resources for diagnosis or treatment but is stable.

Level 4: Requires single resource for diagnosis or treatment, less urgent.

Level 5: Non‐urgent, requires no additional resources beyond the consultation.

(Agency for Healthcare Research and Quality n.d.; Platts‐Mills et al. 2010).

### 2.4 Ethical Considerations

This study was conducted in accordance with the principles of the Declaration of Helsinki and received full ethical approval from the Institutional Review Board (IRB) at Chaim Sheba Medical Center, Tel‐Hashomer. The approval was granted with the reference number 0143‐23‐SMC. Informed consent was waived by the IRB committee due to the retrospective nature of the study and the use of de‐identified patient data to ensure confidentiality and compliance with ethical standards.

### 2.5 Sample Size Calculation

To determine the appropriate sample size for this study, we conducted an a priori power analysis using G*Power software (Kang 2021). Based on previous research on variability in nurse‐scored ESI, we estimated a medium effect size (*f* = 0.25) for differences in ESI scoring between nurses. Assuming a two‐tailed test, an alpha level of 0.05, and a desired statistical power of 0.80, the minimum required sample size was calculated to be 92 participants. To account for potential data loss or exclusions, we rounded up the sample size to 100 participants.

### 2.6 Statistical Analysis

Statistical analyses were conducted using Python version 3.10. For continuous variables, we reported medians with interquartile ranges (IQR) and means with standard deviations (SD); categorical variables were summarised using percentages. A *p*‐value of less than 0.05 was considered statistically significant.

To compare ESI score assignments between GPT‐4 and human evaluators, we employed the Mann–Whitney *U*‐test. Cohen's kappa was also used to evaluate the agreement between each nurse and the senior physician, as well as between GPT‐4 and the senior physician.

### 2.7 Sub‐Analysis of Clinical Features

Sub‐analysis for age relationship with ESI scores was quantified using Spearman's rank correlation coefficient. For gender, differences in ESI scores were evaluated using the Mann–Whitney *U*‐test.

## 3 Results

The cohort (Table 1) comprised 100 adult patients, with a slight majority being male (55%). The median age was 65 years, with patients' inter‐quartile range (IQR) ranging from 48 to 78 years. Vital signs showed a median temperature of 36.7°C, pulse rate at 80 bpm and oxygen saturation at 97%. Blood pressure readings indicated a median systolic pressure of 132.5 mmHg. Pain levels were generally low, with a median score of 3 on a 0–10 scale.

**TABLE 1:** Descriptive statistics of the study sample.

Variable	Median (IQR)
Age	65 (48–78)
Temperature (°C)	36.7 (36.5–37.0)
Pulse (bpm)	80 (71–94)
Systolic BP (mmHg)	132.5 (119.75–153.5)
Saturation (%)	97 (96–98)
Pain (0–10 scale)	3 (1.5–3)
Gender	Male: 55 (55%), female: 45 (45%)

### 3.1 ESI Distribution

**FIGURE 2:**
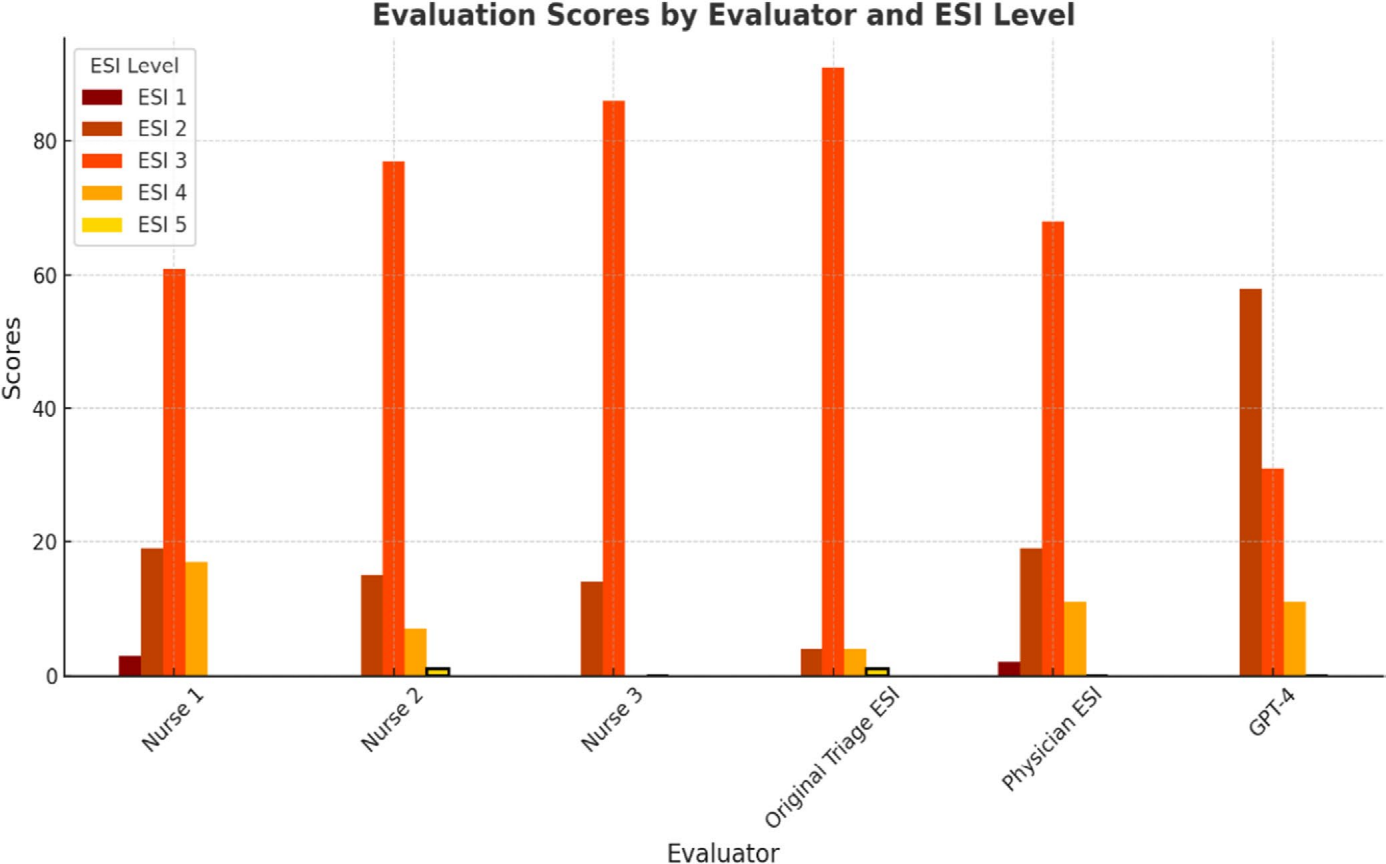
ESI distributions among evaluators. [Colour figure can be viewed at wileyonlinelibrary.com]

Figure 2 displays ESI score distributions assigned by three nurses, the triage system, GPT‐4 and protocol. Most evaluators agreed on a median score of 3.0, indicating uniformity in severity assessment. However, GPT‐4 differed, favouring a lower median of 2.0 (*p* < 0.001), and showcased broader variability. The consistency in scores among human evaluators contrasts with GPT‐4's tendency towards lower severity assignments, highlighting differences between LLMs and human judgement in emergency severity indexing.

Cohen's kappa calculations showed that the agreement between the nurses and the senior physician was relatively higher, with values of 0.30, 0.20 and 0.28 for Nurses 1, 2 and 3 respectively. In contrast, the agreement between GPT‐4 and the senior physician was significantly lower at 0.10.

### 3.2 Sub‐Analyses by Co‐Factors

The analysis of ESI score assignments by gender reveals minimal differences between male and female patients across all evaluators (*p* > 0.05), including GPT‐4 and human evaluators (Appendix 1).

The analysis of age correlations with ESI scores across evaluators (Appendix 2) showed a consistent negative correlation, indicating that older patients tend to receive lower ESI scores (i.e., higher urgency). The strongest negative correlation was observed with GPT‐4 (−0.38). In contrast, the ESI per protocol assignments showed the weakest correlation with age (−0.09).

Table 2 presents a sub‐analysis examining these variations in how the patient vitals were factored into the triage assessments. GPT‐4 demonstrated a notably positive correlation with temperature (0.22) and saturation (0.14), and a significant negative correlation with systolic blood pressure (−0.22).

**TABLE 2:** ESI distributions among evaluators.

Evaluator	ESI 1	ESI 2	ESI 3	ESI 4	ESI 5	Median (IQR)	Mean (SD)
Nurse 1	3	19	61	17	0	3.0 (3–3)	2.92 (0.69)
Nurse 2	0	15	77	7	1	3.0 (3–3)	2.94 (0.51)
Nurse 3	0	14	86	0	0	3.0 (3–3)	2.86 (0.35)
Original triage ESI	0	4	91	4	1	3.0 (3–3)	3.02 (0.35)
GPT‐4	0	58	31	11	0	2.0 (2–3)	2.53 (0.69)
Physician ESI	2	19	68	11	0	3.0 (3–3)	2.88 (0.61)

## 4 Discussion

We examined the assignment of ESI scores using GPT‐4 and compared it to that of senior nurses and an ED physician. A notable finding is GPT‐4's propensity to assign a lower median ESI score of 2, indicating a higher level of urgency, compared to the consistent median score of 3 from human evaluators. This discrepancy suggests that GPT‐4 may overestimate the severity of patients' conditions.

GPT‐4 often frequent assignment level 2 scores show its cautious approach in emergencies, leaning towards over‐triage to avoid underestimating patient conditions. This cautiousness, while preventive, can disrupt emergency department operations, taxing resources and delaying care for critically ill patients, thus necessitating a balance between vigilance and efficient resource use.

Building on previous LLM triage research, like Raita et al.'s study on machine learning for clinical outcome prediction in emergency departments, our analysis of GPT‐4's tendency to assign more level 2 ESI scores offers a nuanced perspective. Unlike Raita et al., who evaluated general accuracy, our focus reveals GPT‐4's specific over‐triage bias (Raita et al. 2019).

The results of our study show similarities to those reported by Zaboli et al. (2024). Both found that while LLMs demonstrated capability for clinical reasoning through complex scenario navigation, they displayed meaningful discrepancies compared to experienced clinicians when directly assessing patient risk stratification and triage prioritisation.

Specifically, Zaboli et al. (2024) observed lower concordance between ChatGPT and nurses for triage code assignment using the Manchester Triage System. Similarly, we found GPT‐4 tended to assign a lower median ESI score than human evaluators in our study, suggesting potential overestimation of patient severity. These parallel findings utilising different triage systems imply LLM limitations transcend any single framework.

A key recurring similarity was inferior predictive performance by LLMs versus clinicians. Zaboli et al. (2024) reported area under the receiver operating characteristic curve values were higher for nurses across important outcomes like 72‐h mortality risk prediction. In our study, GPT‐4 was found to take divergent approaches to triage factors such as patient age and vital signs compared to experienced evaluators. While our methodology incorporated live patient assessment and Zaboli et al. (2024) used recreated vignettes, evaluation of core triage processes in both studies yielded comparable outcomes. This reinforces the broader challenge for LLMs in fully simulating the nuanced judgement of experienced humans, despite demonstration of understanding through complex clinical scenario navigation.

Other studies show GPT‐4 can navigate complex ED scenarios (Haim et al. 2024; Rosen and Saban 2023; Saban and Dubovi 2024). However, previous work found that GPT‐4 struggled with more nuanced clinical decision‐making tasks requiring interpretation of assessment scores. For example, GPT‐4's performance was evaluated in assigning Truelove and Witts scores for cases of ulcerative colitis. This suggests current language models may still have difficulties accurately applying various clinical assessment tools and guidelines to patient presentations, which requires more sophisticated clinical reasoning abilities (Levartovsky et al. 2023). Interestingly, in those studies, as in the current one, GPT‐4 did not perform the same as expert ED personnel. Our research points to GPT‐4's bias in ESI scoring, highlighting the need for LLM that aligns more closely with real‐world clinical demands (Levartovsky et al. 2023).

Several factors may contribute to GPT‐4's behaviour. First, the model's training data and the inherent biases in these datasets can significantly influence its decision‐making process. If GPT‐4 was trained on data where severe outcomes were over‐represented, it might learn to prioritise caution excessively. Second, the LLMs inability to capture the nuanced clinical judgements that experienced nurses apply in real‐time may lead to discrepancies in ESI scoring.

In response to these findings, it is essential to consider the balance between leveraging LLM's capabilities to support emergency care and recognising its current limitations. Future iterations of LLM applications in clinical settings should focus on fine‐tuning these systems to align more closely with practical clinical judgement and decision‐making. This includes improving the LLMs understanding of clinical nuances and developing a more sophisticated assessment of patient severity that mirrors the seasoned insight of human practitioners.

It should be noted that the aim of using LLMs to assign ESI scores is to support, not replace, human triage decisions. The integration of AI can offer consistent triage evaluations, especially during busy times, and provide a second layer of clinical decision support by highlighting critical data that might be missed. This is particularly useful in settings with fewer resources, where experienced triage nurses are scarce.

Our study has several limitations. First, we chose to evaluate GPT‐4 since it is considered state‐of‐the‐art LLM which has been studies extensively in clinical scenarios; however, there are numerous other LLMs that can be further investigated. As only one type of LLM was evaluated here (GPT‐4), performance may differ for other AI architectures trained specifically for clinical decision support tasks. Issues with GPT‐4 overestimating acuity in some cases could potentially be addressed through model optimisations like fine‐tuning on medical corpora. We used a single prompt; a different choice of prompt may have resulted in other results.

Another limitation is observed variability among the participating nurses which should not occur with a standardised triage system. As noted in other research, inter‐rater differences can persist among medical staff despite standardisation efforts, possibly due to individual experience levels, training backgrounds, and unconscious decision‐making tendencies (Sax et al. 2023; Zaboli et al. 2023). Moreover, we used the openAI web‐interface to query GPT‐4; thus, we did not experiment with hyper‐parameters such as temperature. Finally, future studies can explore ensemble methods to augment GPT‐4 with either machine learning models or retrieval augmented generation (RAG) embedding models.

## 5 Conclusion

While GPT‐4 presents a novel approach to emergency patient triage, this study underscores the importance of refining AI tools to better match the complexity and variability of clinical care environments. The tendency of GPT‐4 to overestimate patient severity highlights the need for ongoing assessment and calibration of LLM systems in healthcare to ensure they complement rather than complicate clinical decision‐making processes.

## Conflicts of Interest

The authors declare no conflicts of interest.

## Supporting information


Data S1.

## Data Availability

The data that support the findings of this study are available on request from the corresponding author. The data are not publicly available due to privacy or ethical restrictions.

## Appendix 1 Mean ESI triage scores assigned to male and female patients by different evaluators

### 1.1

**Table:**

Evaluator	Male mean ESI	Female mean ESI
ESI nurse 1	2.95	2.89
ESI nurse 2	2.95	2.93
ESI nurse 3	2.84	2.89
ESI triage	3.02	3.02
GPT‐4	2.58	2.47
ESI per protocol	2.89	2.87

## Appendix 2 Spearman rank correlation between patients' age and ESI triage scores assigned by different evaluators

### 2.1

**Table:**

Evaluator	Age correlation
ESI nurse 1	−0.26
ESI nurse 2	−0.32
ESI nurse 3	−0.15
ESI triage	−0.20
GPT‐4	−0.38
ESI per protocol	−0.09
